# Objectively Measured Built Environments and Cardiovascular Diseases in Middle-Aged and Older Korean Adults

**DOI:** 10.3390/ijerph18041861

**Published:** 2021-02-14

**Authors:** Eun Young Lee, Jungsoon Choi, Sugie Lee, Bo Youl Choi

**Affiliations:** 1 Department of Nursing, Kkottongnae University, Cheongju 28211, Korea; eylee@kkot.ac.kr; 2 Department of Mathematics, Hanyang University, Seoul 04763, Korea; 3 Research Institute for Natural Sciences, Hanyang University, Seoul 04763, Korea; 4 Department of Urban Planning and Engineering, Hanyang University, Seoul 04763, Korea; sugielee@hanyang.ac.kr; 5 Department of Preventive Medicine, College of Medicine, Hanyang University, Seoul 04763, Korea; bychoi@hanyang.ac.kr

**Keywords:** built environment, cardiovascular diseases, hypertension, diabetes, dyslipidemia, stroke, myocardial infarction, angina, middle-aged and older adults, Korea

## Abstract

This study assesses the association between the objectively measured built environment and cardiovascular diseases (CVDs) in 50,741 adults from the Korean Community Health Survey. The CVD outcomes of hypertension, diabetes, dyslipidemia, stroke, and myocardial infarction (MI) or angina were derived from self-reported histories of physician diagnoses. Using ArcGIS software and Korean government databases, this study measured the built environment variables for the 546 administrative areas of Gyeonggi province. A Bayesian spatial multilevel model was performed independently in two age groups (i.e., 40–59 years or ≥60 years). After adjusting for statistical significant individual- and community-level factors with the spatial associations, living far from public transit was associated with an increase in the odds of MI or angina in middle-aged adults, while living in neighborhoods in which fast-food restaurants were concentrated was associated with a decrease in the odds of hypertension and stroke. For adults 60 or older, living farther from public physical-activity (PA) facilities was associated with a 15% increased odds for dyslipidemia, compared with living in neighborhoods nearer to PA facilities. These findings suggest that creating a built environment that provides more opportunities to engage in PA in everyday life should be considered a strategy to reduce the prevalence of CVD.

## 1. Introduction

Cardiovascular disease (CVD) has remained the leading cause of death worldwide, including in the Republic of Korea, despite a marked decrease in incidence rates in recent decades [1]. In 2016, CVD accounted for 31% of all deaths globally [2] and for 15% of all-age, all-cause, disability-adjusted life-years (DALYs) in 2017 [1]. A total of 61,009 Koreans died from CVD in 2018 and it accounted for 20.4% of all deaths [3]. Moreover, DALYs per 100,000 Korean population for CVD were 3475 (11.8%) in 2015 [4]. 

Although individual-level risk factors for CVD have been addressed, neighborhood-level risk factors that can reduce CVD and cardiovascular health disparities are now attracting attention [5]. Because previous studies of neighborhood-level risk factors focused largely on socioeconomic deprivation, social cohesion, air pollution, and traffic noise, little is known about the relationship between the built environment and cardiovascular health [5,6,7]. A systematic review of 18 studies revealed that density of food outlets (restaurants, supermarkets, or grocery stores) and highly walkable environments were associated with blood pressure of neighborhood residents [8]. Additionally, the influence of neighborhood walkability on hypertension and diabetes was confirmed in a meta-analysis of longitudinal studies [9]. Neighborhood walkability as measured by population density, street connectivity, and land-use mix (LUM) can encourage physical activity (PA) and reduce the risk of obesity, diabetes, hypertension, and dyslipidemia [8,9,10,11]. High volume of traffic, road proximity, and dense fast-food restaurants were associated with CVD, including coronary heart disease, stroke, myocardial infarction (MI), and angina [12,13]. Thus, previous studies indicate that PA-favorable neighborhood environment and food environment are related to CVD. 

Although multiple studies have investigated the association between the built environment and cardiovascular health, the effects on hypertension, diabetes, dyslipidemia, stroke, MI, and angina have received much less attention than obesity [5,8,9]. Diverse research on the relationship between built environment and CVD beyond obesity is needed to develop more effective CVD prevention strategies for the population. Additionally, few studies have used specific environmental attributes such as street connectivity, population density, and land-used mix. Most assessed combined environmental measures such as walkability and urban sprawl [8,9]. More research is therefore required on whether specific attributes of the built environment are related to CVD. 

A lack of knowledge on the relationship between the built environment and CVD prompted this study of the relationship between objective measures of built environment and various CVDs including hypertension, diabetes, dyslipidemia, stroke, MI, and angina. 

## 2. Methods

### Study Participants 

Participants in this study were part of the Korean Community Health Survey (KCHS) in 2013 and 2014 [14], an annual nationwide and community-based cross-sectional survey of 230,000 adults aged ≥19 years old. The protocols of the KCHS were approved by the Institutional Review Board of the Korea Centers for Disease Control and Prevention (2013-06EXP-01-3C and 2014-08EXP-09-4C-A) before data collection. Written informed consent was obtained from all participants. Trained interviewers conducted face-to-face computer-assisted individual interviews using a standardized questionnaire in the participants’ homes [14]. The present study used data drawn from the KCHS in Gyeonggi province in 2013 and 2014. The province, which surrounds the South Korean capital of Seoul, represents approximately 10.2% of the total land area and approximately 24% of the total population. Gyeonggi province is a mix of urban and rural areas, consisting of 546 administrative districts in 2013, with an average population of approximately 28,000 residents living in each administrative district. In the current study, we excluded data with incomplete information (*n* = 4387), participants under 40 years of age (*n* = 27,074), and those who were in bed all day and therefore had little involvement in social activities (*n* = 217). A total of 50,741 participants aged 40 years or older were analyzed. 

## 3. Measures

### 3.1. Cardiovascular Disease 

A self-reported history of a physician’s diagnosis of hypertension, diabetes, dyslipidemia, stroke, MI, or angina constituted CVD for the purposes of this study. KCHS collects data using a standardized questionnaire developed by Korea Centers for Disease Control and Prevention [14]. Quality control of the data is conducted every year [14], and reliability and validity of self-reports were assessed [15,16]. Participants who responded “yes” to the item “Have you ever been diagnosed with hypertension by a doctor?” were assigned to the hypertension group. Diabetes was identified by a response of “yes” to the item “Have you ever been diagnosed with diabetes by a doctor?” Dyslipidemia was identified by a response of “yes” to the item “Have you ever been diagnosed with dyslipidemia, including hyperlipidemia, by a doctor?” Stroke was identified by a response of “yes” to the item “Have you ever been diagnosed with stroke by a doctor?” Participants who responded “yes” to “Have you ever been diagnosed with MI or angina?” were assigned to the MI or angina group. 

### 3.2. Built Environment 

This study measured built environment variables using a geographic information system. Metrics included population; density of public physical-activity (PA) facilities, public parks, and fast-food restaurants; proximity to public PA facilities (e.g., athletic or football fields, tennis courts, or swimming pools), parks, and public transit (e.g., subway stations or bus stops); street connectivity; residential density; commercial density; industrial density; and land-use mix (Table 1). Korean government databases (i.e., Population Census 2013, National Public Physical-Activity Facility Database 2013, National Building Database 2013, and Korean Transport Database 2013) and ArcGIS software were used to measure the built environments of the 546 administrative districts (the smallest administrative spatial unit) of Gyeonggi province. 

First, population density was calculated by dividing the total population by the area of urbanized land. This study classified the urbanized areas of each administrative district excluding highland and watershed areas and natural open spaces. The density of PA facilities was the area of PA facilities divided by the population. The density of public parks was expressed as the area of public parks divided by the population. The density of fast-food restaurants was calculated as the number of fast-food restaurants divided by the urbanized area. After forming 100 × 100 m grids in the urbanized areas, proximity to public PA facilities, parks, and public transit was calculated as the average Euclidean distance between the centers of each grid and the nearest destination. Street connectivity was calculated as the number of three- or four-way intersections divided by the urbanized area. Next, this study calculated land-use density measures of residential, commercial, and industrial uses. Residential density was calculated as the residential building floor areas divided by the urbanized area excluding mountains, watershed, and natural open spaces. Commercial and industrial density measures were calculated as the building floor area of each land-use category divided by the urbanized area. This study calculated the land-use mix (LUM) based on floor area of residential, commercial, and industrial buildings [17]. As an entropy index, LUM values close to 1 indicated the highest heterogeneity of land-use. Due to the non-linear effects of the built environment on CVDs, all built-environment variables were categorized into three groups according to tertile distribution. 

### 3.3. Covariates 

Covariates included individual factors such as socio-demographic characteristics, health behaviors, and health status that were obtained from the KCHS. Sex (male or female), age (40–59 years or ≥60 years), education (≤high school diploma or ≥college), household income (<3 million won per month or ≥3 million won per month), job (non-manual job, manual job, or other), living alone, one-person household (no or yes), and residence period (<20 years or ≥20 years) were assessed as the socio-demographic characteristics. Smoking (never, former smoker), alcohol drinking (never, former, or current), sleeping duration (<7 h per day or ≥7 h per day), participation in moderate or vigorous physical activity (no or yes), and level of dietary sodium (high, middle, or low) were assessed. Subjective health (poor or good), perception of stress (no or yes), symptoms of depression (no or yes), and obesity (<25.0 kg/m^2^ or ≥25.0 kg/m^2^) were assessed as health status variables. 

## 4. Statistical Analyses

Statistical analyses were conducted independently in two age groups (40–59 years and ≥ 60 years). We examined the effects of the individual factors on CVD. Conditioning the significant individual factors, we examined the effects of the built environmental factors on CVD. Univariable analyses were performed by using logistic regression models. Multivariable logistic regression models were performed to identify significant individual and built-environment factors [18].

A Bayesian spatial multilevel model was considered to investigate the relationship between the built environment and CVD [18,19,20]. The model was designed to account for complex spatially dependent structures, which mean spatial associations between adjacent geographical areas. The series of models is described in full in Appendix A. Model 1 contained only the intercept; Model 2 contained statistically significant individual factors; Model 3 contained statistically significant built-environmental factors as well as significant individual factors; and Model 4 contained spatially dependent structures, along with significant individual and built-environmental factors, to explain the additional spatial-dependent random variation not captured by neighborhood- and individual-level factors. The spatially dependent component followed a conditional autoregressive distribution [21] that assumed that the random component at a specific area correlated with those in the adjacent neighborhoods. All models included spatially independent random components to account for the spatially independent variation.

In fitting these models, integrated nest Laplace approximation (INLA) as Bayesian estimation methods was performed using the R-INLA package [22] for the computational efficiency. Non-informative priors were considered for all the parameters. The best model was Model 4 based on a deviance information criterion (DIC) [23], which is the goodness-of-fit measure in the Bayesian model comparison (Appendix A). In Model 4, the spatial fraction was computed to determine the extent to which the unexplained spatial variation was associated with geographical location (Appendix A). A spatial fraction close to 1 indicated dominance of the spatial effect [24]. 

## 5. Results

**Figure 1:**
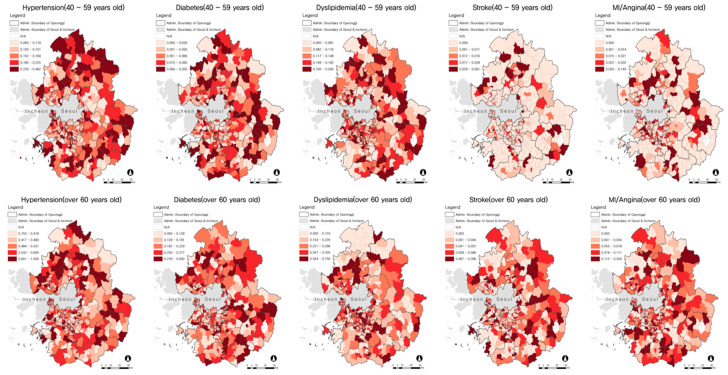
Spatial distribution of prevalence of cardiovascular diseases by age groups.

Table 2 shows the prevalence of CVDs and the general characteristics of the study participants. More than half were female (53.6%), 40–59 years old (65.9%), and had more than a high school diploma (70.2%). Approximately 28.5% of participants had hypertension and approximately 17% had dyslipidemia, followed by 11.3% for diabetes. The prevalence of stroke and MI/angina were approximately 2% and 3.2%, respectively. Figure 1 provides the spatial variations of CVDs by age group (i.e., 40–59 years and ≥60 years). Hypertension, diabetes, and dyslipidemia were more prevalent among those 40–59 years old than among those 60 or older, while stroke and MI/angina were more common in participants 60 years or older. 

The association between CVD and the built environment in adults aged 40–59 years is provided in Table 3. Hypertension was negatively associated with fast-food restaurant density (odds ratio (OR) = 0.91, 95% confidence interval (CI) = 0.84–0.99 for T2 vs. T1). Diabetes was associated with population density, proximity to public park, and residential density, while dyslipidemia was associated with population density, fast-food restaurant density, and proximity to public PA facilities in univariable analysis. However, no built-environment factors significantly influenced diabetes or dyslipidemia in multivariable analysis. Stroke in participants 40–59 years old was negatively associated with fast-food restaurant density (OR = 0.58, 95% CI = 0.41–0.83 for T2 vs. T1; OR = 0.64, 95% CI = 0.44–0.92 for T3 vs. T1). In addition, living in a neighborhood with middle-level distances to public transit was associated with a 36% increase in the odds of diagnosis of MI or angina in adults aged 40–59 compared with those living in a neighborhood with close access to public transit (OR = 1.36, 95% CI = 1.07–1.74 for T2 vs. T1). 

The association between CVDs and the built environment in adults ≥60 years is provided in Table 4. Hypertension, stroke, and MI/angina for those 60 and older were not associated with built-environment factors, while diabetes was associated with LUM (OR = 1.11, 95% CI = 1.01–1.23 for T2 vs. T1; OR = 1.13, 95% CI = 1.02–1.24 for T3 vs. T1). Although dyslipidemia was associated with most of built-environment factors in univariable analysis, the association was statistically significant only for the influence of distance to PA facilities. Living in a neighborhood farther from public PA facilities was associated with a 15% increased odds for dyslipidemia diagnosis in adults 60 or older compared with those living in a neighborhood with close access to public PA facilities (OR = 1.15, 95% CI = 1.01–1.30 for T2 vs. T1) 

## 6. Discussion

The current study assessed the relationship between the objectively measured built environment and various CVDs, including hypertension, diabetes, dyslipidemia, stroke, and MI or angina among middle-aged and older Korean adults. The main finding of this study was that the proximity to public transit was positively associated with MI/angina in adults aged 40–59, while the density of fast-food restaurants was inversely associated with hypertension and stroke. For adults 60 and older, distance to public PA facilities and LUM were significantly associated with dyslipidemia and diabetes, respectively.

These results build on previous findings regarding the relationship between built environments favorable for PA and CVD. The current study found that the proximity to public transit was associated with MI/angina in adults aged 40–59 who actively engaged in social activities. A lack of previous studies on the relationship between public transit and MI/angina precluded any firm conclusions. However, one possible explanation is that the proximity to public transportation encouraged residents to walk to such transport locales [25], which could lead to reduced adiposity and MI/angina. A recent study on the association between public transportation and cardiometabolic health reported that public transportation was associated with modestly lower adiposity rates, because it provided an opportunity to incorporate PA into a journey [26]. A study of commuting modes and CVD mortality involving 394,746 participants who were followed for more than 25 years reported that rail commuters had a 21% lower rate of CVD mortality compared with motor-vehicle commuters [27]. In line with previous studies, our findings support that the proximity to public transit options promotes physically active lifestyles and protects against the development of adiposity and MI/angina in adults aged 40–59. The proximity of PA facilities was significantly associated with dyslipidemia in people aged 60 or older who tend to remain in their neighborhood due to presence of other retirees. Two previous studies of Australian adults reported no association between the built environment and dyslipidemia [28,29]. One study of 78,023 Toronto, Canada residents reported significant differences in the mean high-density lipoprotein levels between the highest and lowest walkability quartiles in adults older than 40; the difference was attributed to a PA effect [10]. The findings in this study expand on previous evidence by adding age-specific associations between the PA-favorable built environments (i.e., proximity of public transit and PA facilities) and certain CVDs (i.e., dyslipidemia and MI/angina). 

The reason why a high density of fast-food restaurants was negatively associated with hypertension and stroke is unclear. Very little is known about the association between the density of fast-food restaurants and hypertension or stroke. A study of more than four million Swedes between 35 and 80 years of age reported a statistically significant association between fast-food restaurants and stroke, although the ORs of the association were not large [12]. In addition, a study from the United States showed a significantly positive association between fast-food restaurants and mortalities related to CVD and stroke [30]. However, a longitudinal study from Australia found no association between the food environment and hypertension [28]. Even a study of 40,398 residents in a rural state in the United States reported an inverse association between fast-food restaurants and CVD health behaviors [31]. Our findings can be explained by a lack of association between proximity to fast-food restaurant and actual fast-food consumption, particularly among middle-aged Asians. Increased age was associated with less frequent consumption of fast-food even considering proximity to and coverage of fast-food restaurants [32]. In addition, spatial distribution data suggest that the prevalence of hypertension and stroke were high in remote areas, which are less urbanized. As a substitute for urbanization, therefore, fast-food restaurants may be related to hypertension and stroke. This unexpected finding raises the need for future replicated studies and longitudinal studies of the association between fast-food restaurants and CVDs. 

The present study found that higher LUM was associated with greater prevalence of diabetes in adults aged 60 or over. Previous studies have supported that high-level LUM encourages participation in physical activity due to better access to local destinations that help reduce obesity and diabetes [33,34,35]. The elderly, who are more sensitive to stress from their residence environments, might be exposed to higher levels of stresses when living in areas with mixed residential, commercial, and industrial land-use, usually located in urban centers [36,37]. In addition, there might confounding factors that were not measured in this study, such as noise and traffic volume [6]. Explanations for the present findings warrant further study on the potential positive and negative effects of LUM on elderly health. 

A major strength of the current study is that it assessed the relationship between the objectively measured built environment and various CVDs. It adjusted for sufficient individual-level covariates in the multilevel framework and considered spatial associations between adjacent geographical areas. The current study has some limitations. First, this study did not allow for a definition of causality association between the built environment and CVD due to a cross-sectional design. Second, although the current study adjusted for the residence period, residents may choose the neighborhoods they live in based on health-related characteristics. Third, because the data on health behaviors and CVDs were limited to self-reported responses in the 2013–2014 KCHS, recall bias cannot be ignored. Further study using medical records is needed. Fourth, although individual-level income was considered in the current study, neighborhood deprivation should be included in the future studies. Finally, various built-environment attributes were including in the current study, but the availability of healthcare facilities were not. 

## 7. Conclusions

The present study supports an age-specific association between the objectively measured built environment (e.g., proximity to public PA facilities and public transit, LUM, and density of fast-food restaurants) and CVDs (hypertension, diabetes, dyslipidemia, stroke, and MI or angina) among middle-aged and older Korean adults. The findings indicate a PA-favorable neighborhood environment with access to public transit options and PA facilities may help reduce CVDs in middle-aged and older adults. The lack of a previous study of whether specific built-environment attributes are related to CVDs implies that similar studies should be conducted. Further examination of the spatial-temporal association is needed to better understand the causality of the relationship between the built environment and CVD.

## Figures and Tables

**Table 1:** The objectively measured built environment.

Variables	Definitions	Data Sources	Mean (±SD)	Min-Max
Population density	Number of people per sq. km of urbanized area	2013 Population Census of Statistics Korea	227.6 (±398.5)	4.2–8067.8
Density of physical activity facilities	Areas of physical activity facility per population	2013 Public Physical Activity Facility Database of the Ministry of Culture, Sports, and Tourism	23,145.9 (±55,203.8)	0.0–513,250.7
Density of public parks	Area of public park per population		451,898.2 (±3008,972.2)	0.0–76,659,160.8
Density of fast-food restaurants	Number of fast-food restaurant per urbanized area	2013 Building Database of the Ministry of Land, Infrastructure and Transport	0.0 (±0.2)	0.0–3.5
Proximity to public physical activity facilities	Average distance between the 100 × 100 m grid center of urbanized areas and public physical activity facilities	2013 Public Physical Activity Facility Database of the Ministry of Culture, Sports, and Tourism	1444.6 (±1216.1)	205.3–9783.6
Proximity to public parks	Average distance between the 100 × 100 m grid center of urbanized areas and public parks		709.3 (± 991.6)	39.9–6183.3
Proximity to public transit	Average distance between the 100 × 100 m grid center of urbanized areas and bus stops or subway stations	2013 Transport Database of the Ministry of Land, Infrastructure and Transport	282.2 (±206.4)	85.1–1751.6
Street connectivity	Number of three- or four-way intersections per urbanized area		0.2 (±0.3)	0.0–2.9
Residential density	Residential building floor areas per urbanized area	2013 Building Database of the Ministry of Land, Infrastructure and Transport	11,734.0 (±53,103.4)	148.7–1,044,944.0
Commercial density	Commercial building floor areas per urbanized area		2750.3 (±7652.3)	49.9–137,521.9
Industrial density	Industrial building floor areas per urbanized area		2305.9 (±6849.8)	49.7–128,049.5
Land-use mix	Residential, commercial, and industrial building floor areas per urbanized area		0.7 (±0.2)	0.0–1.0

**Table 2:** Characteristics of study participants.

Variables	Total	Hypertension	Diabetes	Dyslipidemia	Stroke	MI/Angina
Total, n	50,741 (100)	14,438 (28.5)	5725 (11.3)	8644 (17.0)	1006 (2.0)	1649 (3.2)
Male sex, n (%)	23,549 (46.4)	6923 (47.9)	3013 (52.6)	3717 (43.0)	540 (53.7)	906 (54.9)
40–59 years old, n (%)	33,433 (65.9)	5601 (38.8)	2083 (36.4)	4434 (51.3)	186 (18.5)	418 (25.3)
Education, ≤high school, n (%)	35,632 (70.2)	11,654 (80.7)	4673 (81.6)	6431 (74.4)	861 (85.6)	1344 (81.5)
Household income, <3 million won/month, n (%)	23,482 (46.3)	8672 (60.1)	3580 (62.5)	4649 (53.8)	745 (74.1)	1123 (68.1)
Job, non-manual job, n (%)	10,674 (21.0)	1721 (11.9)	634 (11.1)	1425 (16.5)	45 (4.5)	154 (9.3)
Job, manual job, n (%)	20,264 (39.9)	4977 (34.5)	1865 (32.6)	3043 (35.2)	193 (19.2)	459 (27.8)
Living alone (yes), n (%)	4139 (8.2)	1720 (11.9)	670 (11.7)	954 (11.0)	133 (13.2)	216 (13.1)
Residence period, <20 years, n (%)	27,153 (53.5)	6497 (45.0)	2516 (43.9)	4309 (49.8)	464 (46.1)	736 (44.8)
Former smokers, n (%)	9693 (19.1)	3417 (23.7)	1448 (25.3)	1874 (21.7)	325 (32.3)	505 (30.6)
Current smokers, n (%)	10,509 (20.7)	2414 (16.7)	1123 (19.6)	1479 (17.1)	143 (14.2)	292 (17.7)
Former drinkers, n (%)	6790 (13.4)	2392 (16.6)	1152 (20.1)	1347 (15.6)	279 (27.7)	379 (23.0)
Current drinkers, n (%)	34,807 (68.6)	8572 (59.4)	3119 (54.5)	5452 (63.1)	442 (43.9)	829 (50.3)
Sleeping duration, ≥7 h/day, n (%)	24,906 (49.1)	6974 (48.3)	2897 (50.6)	3938 (45.8)	510 (50.7)	783 (47.5)
Moderate or vigorous physical activity (yes), n (%)	32,589 (64.2)	9204 (63.7)	3537 (61.8)	5460 (63.2)	590 (58.6)	991 (60.1)
Low-sodium diet, n (%)	13,583 (26.8)	4012 (27.8)	1670 (29.2)	2398 (27.7)	299 (29.7)	469 (28.4)
High-sodium diet, n (%)	12,054 (23.8)	3890 (26.9)	1488 (26.0)	2209 (25.6)	270 (26.8)	485 (29.4)
Subjective health (good), n (%)	40,841 (80.5)	9535 (66.0)	3108 (54.3)	5926 (68.6)	335 (33.3)	783 (47.5)
Stress perception (yes), n (%)	13,469 (26.5)	3819 (26.5)	1570 (27.4)	2500 (28.9)	314 (31.2)	453 (27.5)
Depressive symptom (yes), n (%)	3600 (7.1)	1172 (8.1)	549 (9.6)	855 (9.9)	132 (13.1)	197 (11.9)
Obesity (≥25.0 kg/m^2^), n (%)	13,917 (27.4)	5563 (38.5)	2197 (38.4)	3420 (39.6)	337 (33.5)	565 (34.3)
Diabetes (yes), n (%)	5725 (11.3)	3479 (24.1)		2095 (24.2)	294 (29.2)	516 (31.3)
Hypertension (yes), n (%)	14,438 (28.5)				712 (4.9)	972 (6.7)
Dyslipidemia (yes), n (%)	8644 (17.0)				331 (3.8)	713 (8.2)

n (%) = numbers and percentages. MI = myocardial infarction.

**Table 3:** Association between cardiovascular diseases and built environments in adults aged 40–50 years.

Built Environments	Hypertension ^a^		Diabetes ^b^		Dyslipidemia ^c^		Stroke ^d^		MI/angina ^e^	
	Univariable	Multivariable	Univariable	Multivariable	Univariable	Multivariable	Univariable	Multivariable	Univariable	Multivariable
	OR 95%CI	OR 95%CI	OR 95%CI	OR 95%CI	OR 95%CI	OR 95%CI	OR 95%CI	OR 95%CI	OR 95%CI	OR 95%CI
Population density										
T1 (lowest)	1		1	1	1	1	1		1	
T2	0.97 (0.90, 1.05)		**0.83** **(0.74, 0.93)**	0.87 (0.71, 1.06)	**1.10** **(1.01, 1.19)**	1.03 (0.89, 1.21)	0.79 (0.55, 1.15)		0.90 (0.71, 1.16)	
T3 (highest)	0.97 (0.90, 1.05)		0.90 (0.80, 1.01)	0.98 (0.77, 1.26)	1.05 (0.96, 1.14)	0.96 (0.80, 1.15)	0.96 (0.67, 1.39)		0.87 (0.68, 1.12)	
Density of physical activity facilities										
T1 (lowest)	1		1		1		1		1	
T2	0.94 (0.87, 1.01)		0.91 (0.81, 1.03)		1.00 (0.92, 1.09)		0.97 (0.64, 1.44)		1.12 (0.87, 1.43)	
T3 (highest)	1.03 (0.96, 1.11)		1.01 (0.90, 1.12)		0.99 (0.91, 1.07)		1.38 (0.98, 1.96)		1.10 (0.86, 1.39)	
Density of public parks										
T1 (lowest)	1		1		1		1		1	
T2	0.99 (0.92, 1.07)		0.96 (0.86, 1.08)		0.99 (0.91, 1.07)		1.01 (0.71, 1.45)		1.04 (0.82, 1.33)	
T3 (highest)	0.99 (0.91, 1.06)		1.04 (0.93, 1.17)		1.00 (0.92, 1.09)		0.89 (0.62, 1.30)		1.05 (0.82, 1.35)	
Density of fast-food restaurants										
T1 (lowest)	1	1	1		1	1	1	1	1	
T2	**0.91** **(0.84, 0.98)**	**0.91** **(0.84, 0.99)**	0.95 (0.84, 1.06)		1.04 (0.95, 1.13)	1.02 (0.90, 1.16)	**0.58** **(0.41, 0.83)**	**0.58** **(0.41, 0.83)**	1.12 (0.87, 1.44)	
T3 (highest)	**0.92** **(0.85, 1.00)**	0.93 (0.86, 1.02)	0.98 (0.87, 1.11)		**1.10** **(1.01, 1.20)**	1.08 (0.94, 1.25)	**0.64** **(0.44, 0.92)**	**0.64** **(0.44, 0.92)**	1.03 (0.79, 1.35)	
Proximity to public physical activity facilities										
T1 (nearest)	1		1		1	1	1		1	
T2	1.00 (0.93, 1.08)		1.02 (0.92, 1.14)		0.95 (0.88, 1.02)	0.96 (0.86, 1.08)	0.99 (0.70, 1.40)		0.98 (0.78, 1.24)	
T3 (farthest)	1.01 (0.93, 1.09)		1.08 (0.96, 1.22)		**0.91** **(0.84, 0.99)**	0.92 (0.78, 1.08)	1.01 (0.70, 1.47)		1.03 (0.80, 1.33)	
Proximity to public parks										
T1 (nearest)	1		1	1	1		1		1	
T2	0.98 (0.92, 1.06)		0.99 (0.88, 1.11)	1.01 (0.89, 1.13)	0.98 (0.91, 1.06)		0.78 (0.54, 1.12)		1.11 (0.88, 1.41)	
T3 (farthest)	1.03 (0.95, 1.11)		**1.13** **(1.00, 1.26)**	1.05 (0.85, 1.28)	0.93 (0.86, 1.02)		1.05 (0.74, 1.50)		1.11 (0.86, 1.43)	
Proximity to public transit										
T1 (nearest)	1		1		1		1		1	1
T2	1.00 (0.94, 1.08)		1.00 (0.90, 1.11)		1.01 (0.94, 1.09)		0.98 (0.70, 1.39)		**1.41** **(1.11, 1.80)**	**1.36** **(1.07, 1.74)**
T3 (farthest)	0.99 (0.92, 1.07)		1.01 (0.90, 1.14)		0.99 (0.91, 1.08)		0.95 (0.65, 1.38)		1.22 (0.94, 1.60)	1.09 (0.81, 1.46)
Street connectivity										
T1 (lowest)	1		1		1		1		1	1
T2	0.95 (0.88, 1.03)		1.00 (0.89, 1.12)		1.01 (0.93, 1.10)		0.91 (0.64, 1.31)		0.92 (0.72, 1.17)	0.93 (0.72, 1.20)
T3 (highest)	0.95 (0.88, 1.02)		0.94 (0.83, 1.06)		1.06 (0.97, 1.15)		0.83 (0.57, 1.21)		**0.75** **(0.58, 0.97)**	0.77 (0.58, 1.02)
Residential density										
T1 (lowest)	1		1	1	1		1		1	
T2	0.98 (0.91, 1.05)		**0.86** **(0.77, 0.97)**	0.98 (0.79, 122)	1.05 (0.97, 1.14)		0.73 (0.51, 1.05)		0.93 (0.74, 1.18)	
T3 (highest)	0.96 (0.89, 1.04)		0.90 (0.80, 1.01)	0.95 (0.73, 1.22)	1.06 (0.97, 1.16)		0.96 (0.66, 1.38)		0.86 (0.66, 1.11)	
Commercial density										
T1 (lowest)	1		1		1		1		1	
T2	0.97 (0.90, 1.04)		0.91 (0.81, 1.02)		1.06 (0.98, 1.15)		0.79 (0.55, 1.12)		1.01 (0.80, 1.29)	
T3 (highest)	0.99 (0.92, 1.07)		0.90 (0.80, 1.01)		1.04 (0.95, 1.13)		0.86 (0.60, 1.25)		0.87 (0.67, 1.13)	
Industrial density										
T1 (lowest)	1		1		1		1		1	
T2	0.99 (0.92, 1.07)		0.90 (0.81, 1.01)		1.07 (0.99, 1.16)		1.02 (0.71, 1.46)		0.95 (0.75, 1.20)	
T3 (highest)	1.01 (0.93, 1.09)		0.93 (0.83, 1.05)		1.08 (0.99, 1.18)		1.14 (0.79, 1.65)		0.79 (0.61, 1.02)	
Land-use mix										
T1 (lowest)	1	1	1		1		1		1	
T2	1.02 (0.95, 1.10)	1.02 (0.95, 1.10)	0.98 (0.88, 1.10)		1.05 (0.97, 1.14)		0.89 (0.61, 1.30)		0.88 (0.69, 1.13)	
T3 (highest)	**1.08** **(1.00, 1.16)**	1.07 (0.99, 1.16)	1.11 (0.99, 1.25)		1.01 (0.93, 1.10)		1.15 (0.81, 1.65)		1.07 (0.84, 1.36)	
Spatial fraction		0.515		0.473		0.651		0.487		0.518

Univariable and multivariable analyses were adjusted for significant individual variables: (a) adjusted for sex, education, household income, job, living alone, residence period, smoking, sleeping duration, level of dietary sodium, subjective health, perception of stress, symptoms of depression, obesity, and diabetes; (b) adjusted for sex, education, household income, job, living alone, residence period, smoking, alcohol drinking, level of dietary sodium, subjective health, perception of stress, symptoms of depression, and obesity; (c) adjusted for sex, education, household income, job, living alone, residence period, smoking, alcohol drinking, sleeping duration, participation in moderate or vigorous physical activity, level of dietary sodium, subjective health, perception of stress, symptoms of depression, obesity, and diabetes; (d) adjusted for sex, education, household income, job, living alone, residence period, smoking, alcohol drinking, subjective health, perception of stress, symptoms of depression, obesity, diabetes, hypertension, and dyslipidemia; (e) adjusted for sex, education, household income, job, residence period, smoking, alcohol drinking, participation in moderate or vigorous physical activity, level of dietary sodium, subjective health, perception of stress, symptoms of depression, obesity, diabetes, hypertension, and dyslipidemia. Multivariable analysis was performed using a Bayesian spatial multilevel model and adjusted for significant individual variables. MI = myocardial infarction; OR = odds ratio; CI = confidence interval. Significant association in bold as *p* < 0.05 and *p* < 0.001.

**Table 4:** Association between cardiovascular diseases and built environments in adults aged 60 years or older.

Built Environments	Hypertension ^a^		Diabetes ^b^		Dyslipidemia ^c^		Stroke ^d^		MI/angina ^e^	
	Univariable	Multivariable	Univariable	Multivariable	Univariable	Multivariable	Univariable	Multivariable	Univariable	Multivariable
	OR 95%CI	OR 95%CI	OR 95%CI	OR 95%CI	OR 95%CI	OR 95%CI	OR 95%CI	OR 95%CI	OR 95%CI	OR 95%CI
Population density										
T1 (lowest)	1		1		1	1	1	1	1	
T2	0.96 (0.89, 1.03)		0.95 (0.86, 1.03)		**1.12** **(1.02, 1.22)**	0.89 (0.73, 1.10)	**0.79** **(0.66, 0.94)**	0.83 (0.67, 1.04)	0.99 (0.86, 1.14)	
T3 (highest)	0.95 (0.88, 1.03)		0.98 (0.89, 1.08)		**1.20** **(1.10, 1.31)**	0.90 (0.70, 1.16)	1.03 (0.87, 1.23)	1.02 (0.78, 1.32)	0.95 (0.82, 1.10)	
Density of physical activity facilities										
T1 (lowest)	1		1		1		1		1	
T2	0.96 (0.89, 1.03)		1.01 (0.91, 1.11)		1.01 (0.92, 1.10)		1.19 (0.99, 1.44)		0.97 (0.83, 1.13)	
T3 (highest)	1.02 (0.96, 1.10)		1.02 (0.93, 1.11)		0.93 (0.85, 1.01)		1.05 (0.88, 1.25)		0.93 (0.81, 1.07)	
Density of public parks										
T1 (lowest)	1		1		1		1		1	
T2	1.01 (0.93, 1.09)		0.98 (0.89, 1.08)		1.07 (0.98, 1.17)		1.01 (0.84, 1.20)		1.01 (0.88, 1.17)	
T3 (highest)	1.00 (0.93, 1.08)		1.00 (0.91, 1.09)		1.05 (0.96, 1.15)		0.99 (0.83, 1.18)		1.03 (0.89, 1.19)	
Density of fast-food restaurants										
T1 (lowest)	1		1		1	1	1		1	
T2	1.05 (0.97, 1.13)		1.04 (0.95, 1.13)		**1.10** **(1.01, 1.21)**	1.00 (0.88, 1.14)	0.89 (0.75, 1.06)		0.95 (0.82, 1.09)	
T3 (highest)	0.96 (0.88, 1.04)		1.01 (0.92, 1.11)		**1.20** **(1.09, 1.32)**	1.05 (0.90, 1.23)	0.86 (0.72, 1.04)		1.05 (0.91, 1.22)	
Proximity to public physical activity facilities										
T1 (nearest)	1		1		1	1	1		1	
T2	1.08 (1.00, 1.17)		1.04 (0.94, 1.14)		1.02 (0.94, 1.12)	**1.15** **(1.01, 1.30)**	0.88 (0.73, 1.05)		1.08 (0.93, 1.26)	
T3 (farthest)	1.05 (0.97, 1.13)		1.04 (0.94, 1.14)		**0.88** **(0.80, 0.96)**	1.09 (0.90, 1.31)	1.04 (0.87, 1.25)		1.10 (0.95, 1.28)	
Proximity to public parks										
T1 (nearest)	1		1		1	1	1		1	
T2	1.01 (0.93, 1.09)		0.98 (0.89, 1.08)		0.94 (0.86, 1.03)	0.98 (0.86, 1.11)	0.87 (0.72, 1.05)		1.05 (0.90, 1.23)	
T3 (farthest)	1.03 (0.95, 1.11)		1.03 (0.94, 1.13)		**0.82** **(0.75, 0.90)**	0.97 (0.78, 1.20)	1.03 (0.87, 1.23)		1.12 (0.97, 1.30)	
Proximity to public transit										
T1 (nearest)	1		1		1	1	1	1	1	
T2	0.96 (0.89, 1.04)		0.99 (0.90, 1.08)		0.99 (0.90, 1.08)	1.03 (0.89, 1.18)	**0.83** **(0.69, 0.99)**	0.85 (0.68, 1.06)	1.00 (0.86, 1.16)	
T3 (farthest)	1.03 (0.96, 1.12)		0.99 (0.90, 1.09)		**0.87** **(0.79, 0.95)**	0.95 (0.79, 1.14)	0.96 (0.81, 1.15)	0.91 (0.70, 1.18)	1.06 (0.91, 1.23)	
Street connectivity						1				
T1 (lowest)	1		1		1		1	1	1	
T2	1.04 (0.97, 1.12)		0.97 (0.89, 1.06)		**1.12** **(1.03, 1.22)**	1.08 (0.94, 1.23)	**0.81** **(0.68, 0.96)**	0.83 (0.68, 1.01)	0.91 (0.79, 1.05)	
T3 (highest)	1.01 (0.94, 1.10)		0.96 (0.88, 1.06)		**1.20** **(1.10, 1.32)**	1.11 (0.95, 1.30)	0.90 (0.75, 1.07)	0.93 (0.74, 1.16)	0.98 (0.85, 1.14)	
Residential density										
T1 (lowest)	1		1		1	1	1		1	
T2	0.97 (0.91, 1.05)		0.96 (0.88, 1.05)		**1.13** **(1.04, 1.24)**	1.19 (0.88, 1.60)	0.87 (0.74, 1.04)		0.98 (0.85, 1.12)	
T3 (highest)	0.93 (0.86, 1.00)		0.96 (0.89, 1.08)		**1.24** **(1.13, 1.36)**	1.33 (0.94, 1.87)	0.99 (0.82, 1.18)		0.92 (0.79, 1.07)	
Commercial density										
T1 (lowest)	1	1	1		1	1	1		1	
T2	0.96 (0.89, 1.03)	0.97 (0.89, 1.05)	0.95 (0.87, 1.04)		**1.15** **(1.06, 1.26)**	0.82 (0.63, 1.08)	0.92 (0.78, 1.09)		0.99 (0.86, 1.14)	
T3 (highest)	**0.92** **(0.85, 1.00)**	0.94 (0.86, 1.03)	1.02 (0.93, 1.12)		**1.18** **(1.08, 1.29)**	0.81 (0.60, 1.10)	0.94 (0.79, 1.13)		0.94 (0.81, 1.10)	
Industrial density										
T1 (lowest)	1		1		1	1	1		1	
T2	0.99 (0.92, 1.07)		0.99 (0.91, 1.08)		**1.17** **(1.07, 1.27)**	1.05 (0.87, 1.27)	0.85 (0.71, 1.01)		0.99 (0.86, 1.13)	
T3 (highest)	0.93 (0.86, 1.01)		0.95 (0.87, 1.05)		**1.21** **(1.10, 1.32)**	1.05 (0.83, 1.31)	1.06 (0.88, 1.26)		0.96 (0.83, 1.12)	
Land-use mix										
T1 (lowest)	1		1	1	1		1		1	
T2	0.98 (0.91, 1.06)		**1.11** **(1.01, 1.23)**	**1.11** **(1.01, 1.23)**	0.96 (0.88, 1.05)		1.07 (0.89, 1.29)		1.05 (0.91, 1.23)	
T3 (highest)	1.03 (0.96, 1.12)		**1.13** **(1.03, 1.24)**	**1.13** **(1.02, 1.24)**	0.92 (0.84, 1.01)		1.14 (0.95, 1.36)		1.02 (0.88, 1.18)	
Spatial fraction		0.706		0.473		0.938		0.491		0.488

Univariable and multivariable analyses were adjusted for significant individual variables: (a) adjusted for sex, education, household income, job, living alone, residence period, smoking, alcohol drinking, participation in moderate or vigorous physical activity, level of dietary sodium, subjective health, perception of stress, symptoms of depression, obesity, and diabetes; (b) adjusted for sex, education, household income, job, residence period, smoking, alcohol drinking, sleeping duration, participation in moderate or vigorous physical activity, level of dietary sodium, subjective health, perception of stress, symptoms of depression, and obesity; (c) adjusted for sex, education, living alone, residence period, smoking, sleeping duration, subjective health, perception of stress, symptoms of depression, obesity, and diabetes; (d) adjusted for sex, household income, job, smoking, alcohol drinking, participation in moderate or vigorous physical activity, subjective health, perception of stress, symptoms of depression, obesity, diabetes, hypertension, and dyslipidemia; (e) adjusted for sex, household income, smoking, alcohol drinking, participation in moderate or vigorous physical activity, subjective health, perception of stress, symptoms of depression, obesity, diabetes, hypertension, and dyslipidemia. Multivariable analysis was performed using a Bayesian spatial multilevel model and adjusted for significant individual variables. MI = myocardial infarction; OR = odds ratio; CI = confidence interval. Significant association in bold as *p* < 0.05 and *p* < 0.001.

## Data Availability

Availability of KCHS data: https://chs.cdc.go.kr/chs/index.do (accessed on 18 January 2021).

## Appendix A

Bayesian spatial multilevel model

We assume $y_{ki}$ is the binary CVD outcome of individual $i\;\left(=1,\ldots ,n_{k}\right)$ at neighborhood k (=1,…,N), following a Bernoulli distribution with the probability having CVDs, $p_{ki}$ (Equations (A1) and (A2)):

(A1) $$y_{ki}\sim Bernoulli\left(p_{ki}\right),$$

(A2) $$logit\left(p_{ki}\right)=\log\left(\frac{p_{ki}}{1-p_{ki}}\right)=\psi_{ki}.$$

In this study, each model specified $\psi_{ki}$ as follows.

(Model 1) $\psi_{ki}=\alpha +u_{k}$

(Model 2) $\psi_{ki}=\alpha +X_{ki}^{\prime }\beta +u_{k}$

(Model 3) $\psi_{ki}=\alpha +X_{ki}^{\prime }\beta +E_{k}^{\prime }\gamma +u_{k}$

(Model 4) $\psi_{ki}=\alpha +X_{ki}^{\prime }\beta +E_{k}^{\prime }\gamma +u_{k}+v_{k}$,

where α is an intercept, $X_{ki}^{}$ are individual factors, and $E_{k}^{}$ are built-environmental factors with the corresponding coefficients β and γ, respectively. The random component $u_{k}\sim N\left(0,\;\sigma_{u}^{2}\right)$ explains the spatially uncorrelated effects of spatially independent variation. The random component $v_{k}$ explains the spatially correlated effects based on a conditional autoregressive distribution [10] (Equation (A3)):

(A3) $$v_{k}|v_{j,j\neq k\;}\sim \;N\left(\underset{j}{\sum }\frac{w_{kj}v_{j}}{w_{k+}},\;\frac{\sigma_{v}^{2}}{w_{k+}}\right),\;j,k=1,2,\ldots ,\;N.$$

The neighborhood information $w_{jk}$ is 1 when the jth and kth regions are adjacent; otherwise, $w_{jk}$=0. The number of the neighboring regions for the kth region is defined by $w_{k+}=\sum w_{kj}$. The intercept α and fixed parameters β and γ are assigned non-informative prior values, N(0,10000). For the variance parameters $\sigma_{v}^{2}$ and $\sigma_{u}^{2}$, the default priors in R-INLA [11] are used as $\log\left(1/\sigma_{v}^{2}\right)$, $\log\left(1/\sigma_{u}^{2}\right)\sim$logGamma(1,0.0005).

For the goodness-of-fit measure, the deviance information criterion (DIC) is considered as (Equation (A4)):

(A4) $$DIC=pD+\bar{D\left(\theta \right)},$$

where the vector of parameters is θ. The posterior mean of deviance is $\bar{D\left(\theta \right)}$, D(θ)=−2log(y|θ), and the model complexity is $pD=\bar{D\left(\theta \right)}-D\left(\hat{\theta }\right)$, and the posterior mean is $\theta ,\;\hat{\theta }$. A smaller DIC value indicates better model fit.

In Model 4′s spatially uncorrelated and correlated components, the spatial fraction is defined by (Equation (A5)):

(A5) $$SF=\frac{\sigma_{v}^{2}}{\sigma_{u}^{2}+\sigma_{v}^{2}}.$$
